# Apex-to-Cupola Distance Following VATS Predicts Recurrence in Patients With Primary Spontaneous Pneumothorax

**DOI:** 10.1097/MD.0000000000001509

**Published:** 2015-09-18

**Authors:** Jia-Ming Chang, Wu-Wei Lai, Yi-Ting Yen, Yau-Lin Tseng, Ying-Yuan Chen, Ming-Ho Wu, Wei Chen, Richard W. Light

**Affiliations:** From the Department of Surgery, Division of Thoracic Surgery, Chia-Yi Christian Hospital, Chia-Yi; (JMC); Department of Surgery, Division of Thoracic Surgery, National Cheng Kung University Hospital, Tainan; (WWL, YTY, YLT, YYC); Department of Surgery, Division of Thoracic Surgery, Tainan Municipal Hospital, Tainan; (MHW); Division of Pulmonary and Critical Care Medicine, Chiayi Christian Hospital, and Department of Respiratory Therapy, China Medical University, Taiwan; (WC); and Division of Allergy, Pulmonary and Critical Care Medicine, Vanderbilt University Medical Center, Nashville, TN (RWL).

## Abstract

Our study sought to determine whether the size of the residual apical pleural space in young patients with primary spontaneous pneumothorax (PSP) following video-assisted thoracoscopic surgery is associated with the risk of recurrence.

We retrospectively reviewed patients (≤30 years’ old) with primary spontaneous pneumothorax following thoracoscopic surgery (2002–2010) in a university-affiliated hospital. The size of residual apical pleural space was estimated by measuring the apex-to-cupola distance on a postoperative chest radiograph at 2 time windows: first between postoperative day (POD) 0 and 3, and second between POD 4 and 14.

A total of 149 patients were enrolled with a median follow-up of 11.2 months (interquartile range, 0.95–29.5 months), of whom 141 (94.6%) were male with a mean age of 20 years. The postoperative recurrence rate was 11.4%. Comparing the characteristics between the patients with and without recurrent pneumothorax, the patients with recurrence were younger (18.2 + 2.4 vs 20.7 + 3.7 years, *P* = 0.008), with a lower rate of pleurodesis (35% vs1 69%, *P* = 0.037), longer apex-to-cupola distance at POD 0 to 3 (22.41 ± 19.56 vs 10.07 ± 10.83 mm, *P* < 0.001) and POD 4 to 14 (11.82 ± 9.75 vs 5.54 ± 8.38 mm, *P* = 0.005) than the patients without recurrence. In a multivariate logistic regression model for recurrent pneumothorax, age <18 years (*P* = 0.026, odds ratio [OR]: 4.694), apex-to-cupola distance at POD 0 to 3 >10 mm (*P* = 0.027, OR: 5.319), and no pleurodesis during VATS (*P* = 0.022, OR: 5.042) were independent risk factors for recurrent pneumothorax.

The recurrence rate was not low (11.4%) in young patients with PSP following VATS. Residual apical pleural space with apex-to-cupola distance of 10 mm or greater at POD 0 to 3, younger age, and no pleurodesis would increase postoperative recurrence of primary spontaneous pneumothorax.

## INTRODUCTION

Primary spontaneous pneumothorax (PSP) mostly occurs in healthy adolescents and young adults without prior lung disease.^1^ It is a significant global health problem, with a reported incidence of 18 to 28/100,000 cases per annum for men and 1.2 to 6/100,000 for women,^2^ and is notorious for a high recurrence rate (17%∼52%) after the first episode of PSP with conservative treatment,^1,3–5^ and even progressively higher after the second or third episode.^1,6^ The options for the management of PSP are mainly based on the symptoms of the patients and size of pneumothorax. In current opinion, minimally invasive video-assisted thoracoscopic surgery (VATS) is considered effective for treatment of PSP.^7^ Further, VATS combined with chemical or mechanical pleurodesis has been shown to substantially reduce the risk of recurrence in long-term follow-up of patients with PSP,^8^ yet some studies have reported a relatively high recurrence rate, up to 10%∼16%.^9,10^

Residual pneumothorax after chest tube removal was frequently observed in surgical series with estimated rate of 15% to 20% for major lung resections of lobectomy or bi-loboectomy,^11,12^ and 7% to 23% for pneumothorax,^11,13,14^ and only a quarter of these required active treatment.^15^ Presence of the residual apical pleural space (RAPS) on chest radiography before discharge was once proposed to increase the risk of recurrence following surgery for pneumothorax.^16^ However, it is not known whether the size of the RAPS is truly associated with the postoperative recurrence. Our study aimed to investigate whether the size of RAPS by measurement of the apex-to-cupola distance would increase the risk of recurrence following VATS in primarily young patients with PSP.

## MATERIALS AND METHODS

We conducted this retrospective study on patients who underwent VATS for PSP at National Cheng Kung University Hospital during March 2002 to June 2010. The study was reviewed and approved by the Institutional Ethical Committee of the hospital, and individual patient consent was waived. The enrollment criteria were patients of 30 years old or younger, without known underlying lung diseases, and with perioperative chest radiographs.

Clinical demographics, laboratory data, and operative findings were obtained. Chest radiography was performed before and after surgery in all patients. Preoperative pneumothorax severity was assessed using Light index score with the following equations as described: 100−[(average diameter of lung^3^/average diameter of hemithorax^3^) × 100], measured at hilum level.^17,18^ All operations were carried out in a thoracoscopic fashion using 2 to 3 ports. Routine wedge resection would be performed with endo-staplers if blebs or emphysematous bullae were visualized intraoperatively; if no obvious blebs or emphysematous bullae were found, stapled wedge resection at apex of upper lobes would still be undertaken for evidence showing beneficial effect on recurrence prevention.^19^ Pleurodesis was not a routine in the early period of our study but eventually became standard procedure in our institute. Mechanical pleurodesis was performed intraoperatively using pleural abrasion; and chemical pleurodesis was performed intraoperatively using Talc poudrage (5 g) or minocycline slurry (400 mg minocycline in 100 mL normal saline), or postoperatively and additionally at bedside through the chest drainage tubes with minocycline.

### Postoperative Follow-Up

**FIGURE 1:**
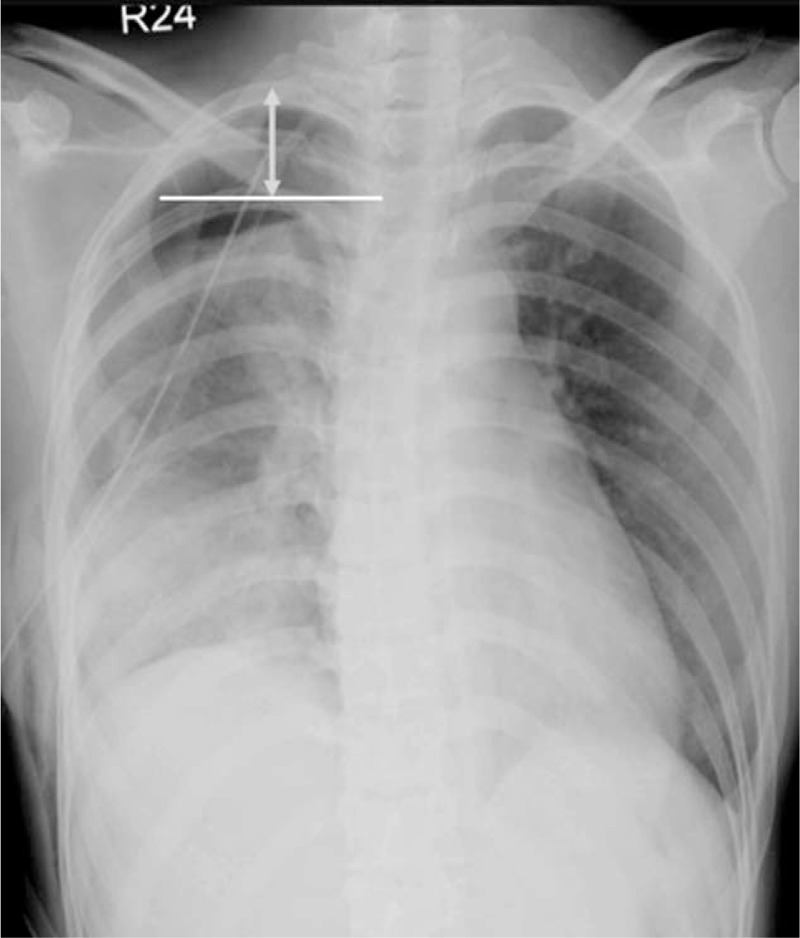
Residual apical pleural space was determined by measuring the lung apex-to-cupola distance (double-headed arrow), which is the vertical distance from the 1st costovertebral joint to the tip of apical lung.

After surgery, follow-up for lung expansion was obtained with standard erect posterioanterior chest X-rays (CXRs) serially until discharge from the hospital and was also obtained in the outpatient clinic. All the procedures of CXR test were according to standard protocol.^20^ The chest tube management was mainly under water-seal and occasional with suction (−10 to −20 cmH_2_O) if air-leak and compromised lung expansion were present. The chest tubes were removed after air-leaks had been sealed. The extent of postoperative lung expansion, as inverse reflection on the size of RAPS, was estimated by measurement of the “apex-to-cupola distance,” also adopted by the American College of Chest Physicians (ACCP) in the guideline for measurement of volume of a pneumothorax.^21^ The apex-to-cupola distance was measured as the vertical distance from the 1^st^ costovertebral joint to the tip of the lung apex as shown in Figure 1.^21^ As this is a retrospective study, the patients did not receive postoperative chest radiography on a uniform schedule. Thus, we measured the apex-to-cupola distance at 2 time windows; first between postoperative day 0 to day 3 (POD0–3), and second between postoperative day 4 and day 14 (POD4–14), regardless of the presence or absence of chest drains. If >1 chest film were available during single time window, only the shortest distance was used for analysis. An experienced radiologist performed all the radiographic measurements. After discharge from the hospital, patients received follow-up in the outpatient department, and the patients were instructed to return to hospital if they experienced any symptoms of pneumothorax such as chest pain or dyspnea. The end-point of this study: recurrence, was defined as another new episode of radiophraphically documented pneumothorax in the same pleural cavity after complete resolution of prior event.

### Statistical Analysis

Continuous data are presented as means ± standard deviation, and differences between continuous variables were measured by the 2-tailed Student *t* test. Categorical data are presented as counts with percentages, and differences were analyzed using the *χ*^2^ or Fisher exact tests. A *P* value <0.05 was taken to indicate statistical significance. Univariate and multiple logistic regression analyses were used to explore the risk factors for the recurrence of pneumothorax. A cut-off value of apex-to-cupola distance was generated using the area under receiver-operating characteristic (ROC) curve method. All data analyses were performed with SPSS software (SPSS 21.0 for Windows, SPSS Inc, Chicago, IL).

## RESULTS

During March 2002 to June 2010, a total of 295 patients underwent VATS for PSP in our institute, and finally 149 consecutive patients were eligible for enrollment in the study with a mean follow-up duration of 19.2 months, (median: 11.2 months; interquartile range: 0.95–29.5 months). Of the 149 patients, 141 (94.6%) were male with a mean age of 20 years (median: 20 years; interquartile range: 18–22 years). The laterality of pneumothorax was almost equal, with the right side accounting for 49.7% (n = 74) and the left side 50.3% (n = 75). Surgical indications included: ipsilateral recurrence in 51 patients (34.2%), persistent air-leak >3 days in 33 cases (22.1%), contralateral recurrence in 32 cases (21.5%), hemopneumothorax in 17 cases (11.4%), conservative treatment failure in 15 cases (10.1%), and totally collapsed lung in 7 cases (4.7%) (6 patients had multiple indications). Wedge resection was performed at single upper lobe in 113 cases (75.8%) and multiple lobes at 36 cases (24.2%). Overall, pleurodesis was performed in 89 patients (59.7%), including 68 intraoperative mechanical + chemical pleurodesis (45.6%), 6 intraoperative mechanical pleurodesis (2.7%), 16 intraoperative chemical pleurodesis (10.7%), and 2 postoperative bedside additional pleurodesis (1.3%) as a reaction to ongoing air leak. Prolonged air-leak (>5 days) occurred in 12 (8.1%) patients. All were managed conservatively with prolonged indwelling chest tube with suction in 5 patients (1 with additional bedside chemical pleurodesis) or without suction in 7 patients. There were no complications requiring reoperation and no surgical mortality in this study. Postoperative recurrence occurred in 17 patients (11.4%) with a mean recurrence interval of 12.1 + 18.6 months (median: 2.9 months; interquartile range: 1.5–18.2 months). Management after recurrence for the patients was as followed: reoperation in 8 cases (47%), all without further episodes; chest tube thoracostomy with chemical pleurdesis in 4 cases (23.5%), all without further episodes; chest tube thoracostomy in 2 cases (11.8%), in 1 case requiring additional chemical pleurodesis at the 2nd recurrence; observation only in 3 cases (17.6%), in 1 case, a total of 3 minor recurrences occurred (Table 1).

**TABLE 1:**
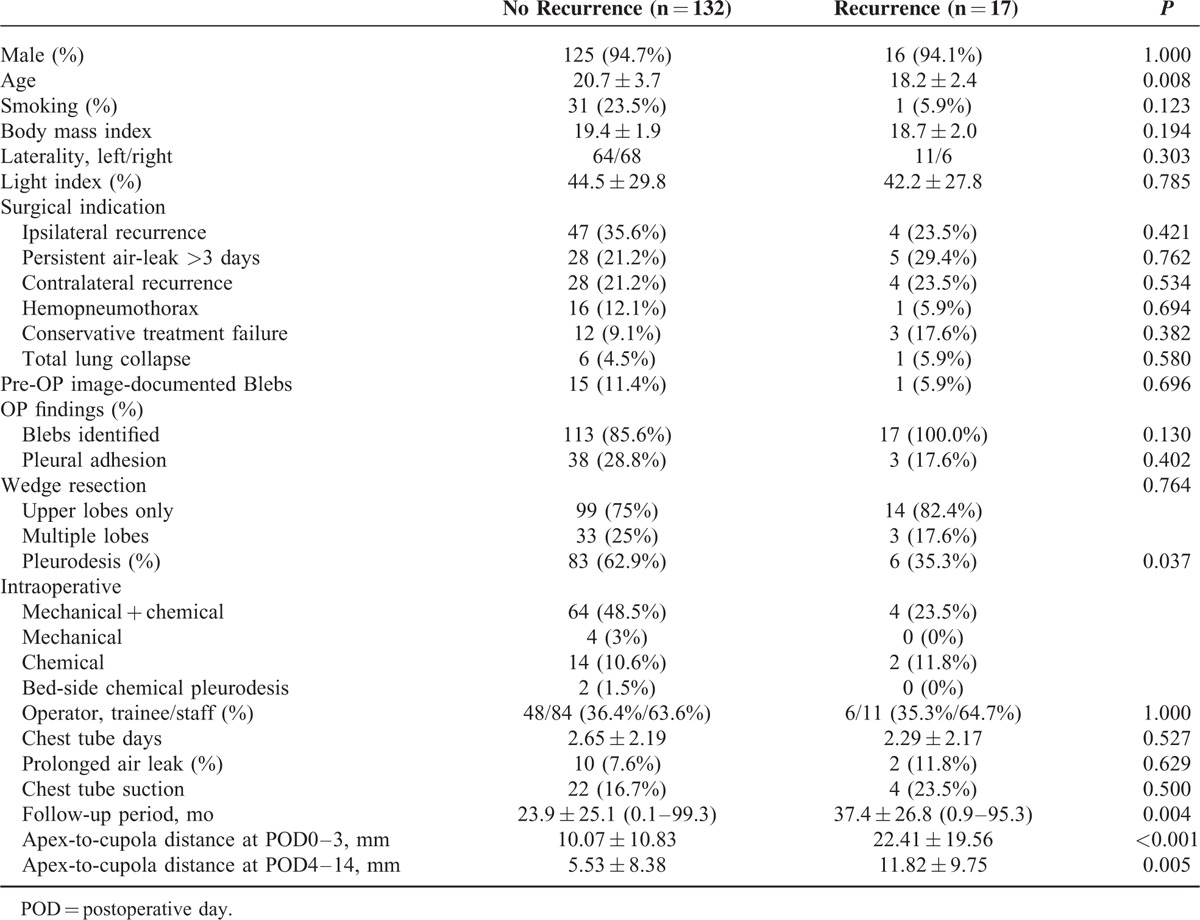
Clinical Characteristics and Apex-To-Cupola Distance Between the Patients With and Without Recurrent Pneumothorax

Comparing the characteristics between the PSP patients with and without recurrence of pneumothorax, those with recurrence were younger than those without recurrence (18.2 + 2.4 vs 20.7 + 3.7 years, *P* = 0.008) (Table 1). There were no significant differences in smoking history, body mass index (median: 19.3; interquartile 18.0–20.7), disease laterality, preoperative Light index of the pneumothorax severity (median: 38.5; interquartile 17.8–69.3). Intraoperative findings of confirmed blebs, pleural adhesion, or wedge resection at single or multiple lobes also showed no influence on recurrence. Primary operators of trainee or staff, chest tube indwelling time, chest tube suction or not, and postoperative prolonged air-leak showed no significant differences between the 2 groups. The follow-up period was significantly longer in the recurrence group (37.4 ± 26.8 to 23.9 ± 25.1 months, *P* = 0.004). The median of apex-to-cupola distance at POD0–3 was 8 mm (interquartile 3–15 mm) and 2 mm (interquartile: 0.0–9.0 mm) at POD4–14 among the all enrolled patients (n = 149). Comparing the RAPS between the 2 groups as measured by the apex-to-cupola distance, the patients with recurrence had a significant longer distance than the patients without recurrence at POD0–3 (22.41 ± 19.56 vs 10.07 ± 10.83 mm, *P* < 0.001) and POD4–14 (11.82 ± 9.75 vs 5.54 ± 8.38 mm, *P* = 0.005).

To further understand whether RAPS is influenced by pleurodesis, we performed subgroup analysis. We used the area under ROC curve method to obtain that 10�mm would have the best sensitivity and specificity (POD0–3: sensitivity 0.7059, specificity 0.5985) as a cut-off value for the apex-to-cupola distance. The patients with recurrence had a significant higher rate of apex-to-cupola distance >10 mm at POD0–3 (82.4% vs 44.7%, *P* = 0.004) and POD4–14 (47.1% vs 20.5%, *P* = 0.029) in all enrolled patients (n = 149) and also in the non-pleurodesis group with POD0–3 (81.8% vs 32.7%, *P* *=* 0.005) and POD4–14 (36.4% vs 8.2%, *P* = 0.031) (n = 60) (Table 2). In the pleurodesis group patients (n = 89), apex-to-cupola distance >10 mm at POD0–3 showed no significance (83.3% vs 51.8%, *P* = 0.212) and at POD4–14 only borderline significance (66.7% vs 27.7%, *P* *=* 0.066) to postoperative recurrence of PSP.

**TABLE 2:**
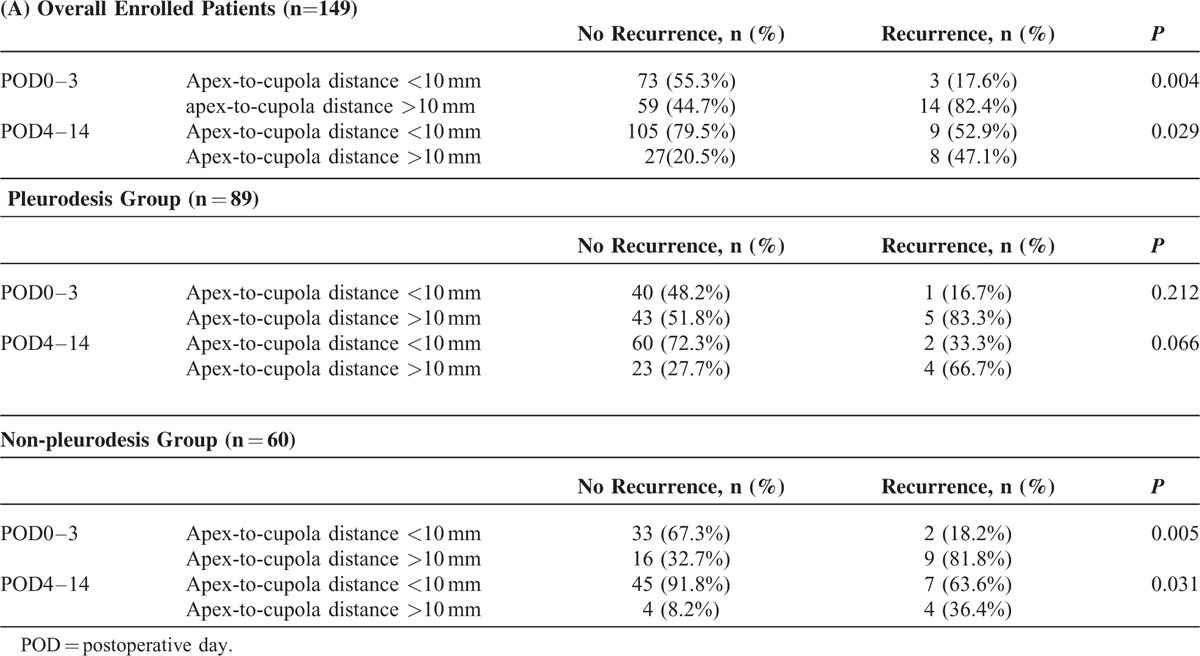
Recurrent Pneumothorax at POD0-3 and POD4-14 in Overall Enrolled Patients (n�=�149), Pleurodesis Group (n�=�89), and Non-Pleurodesis Group (n�=�60)

To further identify the independent risk factors for postoperative recurrence pneumothorax in all enrolled patients, we conducted univariate and multivariate logistic regression analysis for recurrence (Table 3). Only 3 factors with significance (*P* < 0.05) in the univariate analysis would be allowed to enter multivariarte logistic regression. The result showed that younger age (*P* = 0.008, odds ratio [OR] 0.72 [0.56–0.92]), apex-to-cupola distance in POD0–3 >10 mm (*P* = 0.002, OR 9.55 [2.27–40.2]), and no pleurodesis (*P* *=* 0.004, OR 0.16 [0.04–0.54]) were predictors for recurrence of pneumothorax in all 149 patients with PSP following VATS. Nagelkerke R-square was 0.3241 in both univariate and multivariate analysis.

**TABLE 3:**
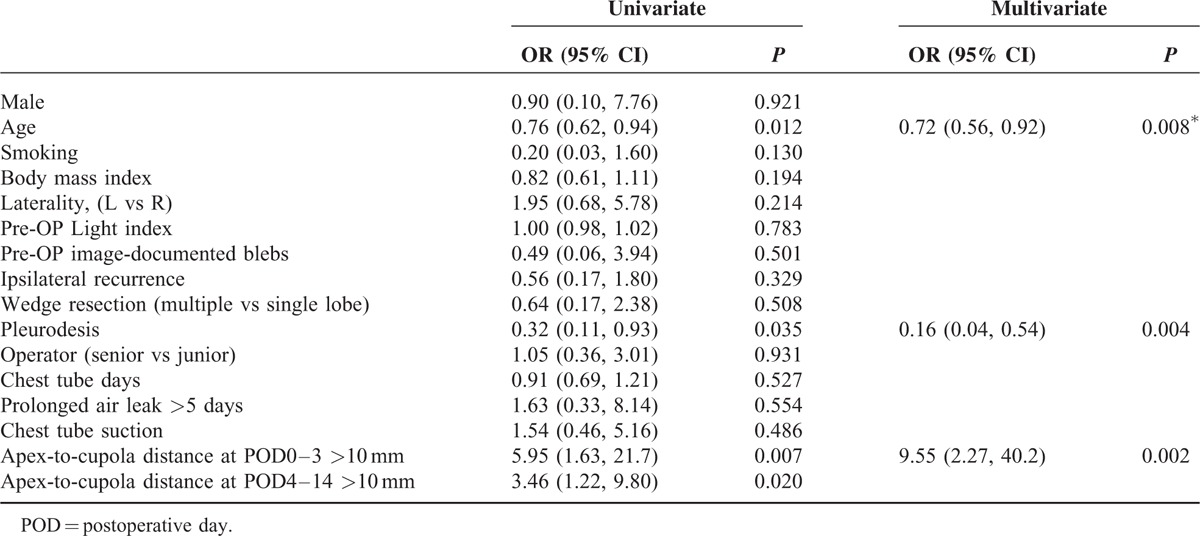
Univariate and Multivariate Logistic Regression Analysis for Predictors of Postoperative Recurrence of Primary Spontaneous Pneumothorax in all 149 Patients

## DISCUSSION

To the best of our knowledge, this is the first study to investigate the correlation between postoperative RAPS, which is determined by measuring the apex-to-cupola distance on a chest radiograph, and the recurrence of pneumothorax in young patients (<30 years’ old) with PSP following VATS. Postoperative recurrence was found in 17 patients (11.4%). Comparing the characteristics between the PSP patients with and without recurrence of pneumothorax, those with recurrent pneumothorax were younger, had a lower rate of pleurodesis during VATS, and a longer apex-to-cupola distance at POD0–3 and POD4–14 than the patients without recurrence. In a multivariate regression model for recurrence, younger age, apex-to-cupola distance at POD0–3 of >10 mm, and no pleurodesis during VATS were independent risk factors for recurrence of pneumothorax in all of the enrolled patients with PSP following VATS.

Reviewing the literature, several factors have been shown to be associated with the postoperative recurrence in patients with PSP, including young age,^22,23^ current smoker,^24^ missed or incomplete bullectomy,^22^ and prolonged postoperative air-leak.^25^ Subpleural blebs and bullae are commonly found in the lung apices during thoracoscopy.^17^ Image documentation of blebs or bullae was proposed by some authors to be significantly related to the ipsilateral PSP recurrence^26,27^; however, the findings would not alter the treatment course and further, almost no evidence showing its correlation to postoperative recurrence of PSP. In our series, the surveillance rate for blebs using preop CT scan is quite low, occurring in only 17 (11.4%) patients; hence, the preop image-documented blebs were not included in our logistic regression for the high possibility of underestimation. In a previous study, younger age was found to be a risk factor for reoperation due to prolonged air leakage or recurrent pneumothorax in patients with spontaneous pneumothorax,^22^ which is compatible with our analysis; however, the mechanism for this is currently unknown. Prolonged postoperative air-leak was also proposed to be associated with recurrence of postoperative pneumothorax recurrence.^25^ However, this was not the case in our series.

It has been widely accepted that 2 key objectives for surgical prevention of pneumothorax recurrence count. The first is to resect any visible bullae or blebs on the visceral pleura. However, the procedure alone was debated to be effective enough; hence, the second to create a symphysis between the 2 opposing pleural surfaces as an additional means is rather crucial.^7,23^ Cumulative evidence had shown that VATS bullectomy combined with mechanical or chemical pleurodesis is effective in preventing the recurrence of pneumothorax,^28^ with a low complication rate and only minor pulmonary function changes at 1 year.^29^ The importance of pleurodesis was further stressed by some reports showing the increasing risk of postoperative recurrent pneumothorax due to incomplete resection of bullae^10^ or bulla regrowth along staple-lines,^30^ and the pleural porosity theory proposed by Noppen et al as a remaining etiology of PSP;^31^ in both, the recurrence could be lowered by pleurodesis.^23,32^ Our study supported that pleurodesis is an independent risk factor in multivariate analysis for the prevention of postoperative pneumothorax recurrence. The majority of our pleurodesis method, a combination of intraoperative mechanical pleurodesis (pleural abrasion) with chemical pleurodesis (by Talc poudrage or minocycline slurry), is a process of evolution and inspiration by the prospective randomized trial by Chen et al^33^ in 2006 showing that additional minocycline pleurodesis could significantly lower the recurrence rate after VATS in PSP but with increased immediate chest pain. We performed both intraoperatively to reduce patient pain and possible recurrence. However, its efficacy and possible advantages over other pleurodesis methods mandate further investigation.

To allow good pleural symphysis to develop, excellent lung expansion must be present, hence minimum residual pleural space to maximum visceral and parietal pleural contact. In 2007, Gaunt et al^16^ was the first and probably the only one in the literature to mention that the presence of residual apical space on chest radiography after surgery increased the risk of recurrent pneumothorax (hazard ratio 3.1). However, his conclusion is based only on univariate analysis for both primary and secondary pneumothoraxes, and no quantitative measurement was made regarding the severity of RAPS. Our study is the first to investigate the correlation between postoperative recurrence of PSP and the post-operative RAPS, which is estimated quantitatively with the ease of chest radiograph apex-to-cupola distance measurement (adopted by the ACCP in the spontaneous pneumothorax management guideline).^12^ The Light index score used in our preoperative evaluation is based on the proportional correlation between the lung and the hemithorax. Its accuracy in particularly smaller pneumothorax remained questionable^17^ and therefore not suitable for RAPS measurement. In our analysis, longer apex-to-cupola distance at POD0–3 and POD4–14 significantly increased the risk of postoperative recurrence of PSP in univariate logistic regression. But in multivariate analysis, only apex-to-cupola distance at POD0–3 reached statistical significance (apex-to-cupola distance at POD4–14 was not chosen into multivariate logistic regression due to multicollinearity concern, we allowed only age, pleurodesis, and apex-to-cupola distance at POD0–3 >10 mm for multivariate analysis for upper limit of 2 binary variables). Another factor that should be considered is the possible correlation of the size of the RAPS to the volume of wedge-resected lung. However, it is quite difficult to assess the resected lung volume, especially on a retrospective basis, we took alternative semiquantitative grouping with wedge resection at single (upper) lobe or multiple lobes. Its correlation to recurrence is irrelevant. Its correlation to the size of RAPS is also insignificant. Taken together, successful prevention of recurrence requires 3 elements, including identifying apical blebs, pleurodesis during the operation, and attempt to expand the lung postoperatively. Therefore, if one of these procedures is not good enough, it may cause larger RAPS and subsequently higher recurrence rate.

There are several limitations to this study. First, this is a retrospective study over a long period of time during which the management for PSP has evolved. In addition, the practice of postoperative radiography follow-up time table was not uniform; pleurodesis modality and chest tube management also differed. Second, the sample size of this study is relatively small and the results are only from 1 center. Nevertheless, our study revealed the potential association of the impaired lung expansion and the postoperative recurrence of PSP. The result brings our attention to optimizing lung expansion in the early postoperative days by ways of chest drainage efficacy adjustment (chest tube size adjustment, suction use, or just prolonged indwelling), additional pleurodesis, or even possible division of inferior pulmonary ligament to facilitate the lung float. The clinical benefit would outweigh just a cut-off value, relying on future large-scale, prospective randomized studies for further validation and investigation.

In conclusion, the postoperative recurrence rate was 11.4% in our patients with PSP. The risk factors for recurrent pneumothorax following VATS included younger age, large residual apical pleural space with apex-to-cupola distance on POD0−3 of >10 mm, and no pleurodesis.
